# Pathways to Endocrine Therapy Resistance in Breast Cancer

**DOI:** 10.3389/fendo.2019.00573

**Published:** 2019-08-21

**Authors:** Md. Moquitul Haque, Kartiki V. Desai

**Affiliations:** Department of Cancer Genomics and Epigenomics, National Institute of Biomedical Genomics, Kalyani, India

**Keywords:** tamoxifen, estrogen receptor, genomic rearrangements, receptor tyrosine kinases, mutations

## Abstract

Breast cancers with positive expression of Estrogen Receptor (ER+) are treated with anti-hormone/endocrine therapy which targets the activity of the receptor, the half-life of the receptor or the availability of estrogen. This has significantly decreased mortality in women with ER+ breast cancer, however, about 25–30% of treated women run the risk or recurrence due to either intrinsic or acquired resistance to endocrine therapies. While ER itself is a predictor of response to such therapies, there exists a need to find more biomarkers and novel targets to treat resistant tumors. In this review, we summarize the known mechanisms and describe the ability of genomics in unraveling rare mutations and gene rearrangements that may impact the development of resistance and therefore treatment of ER+ breast cancer in the near future.

## Introduction

According to GLOBOCAN 2018 around 2.1 million female breast cancer cases will be newly diagnosed in the year 2019 (1). Historically, breast cancer was classified on the basis of histopathology but with the advent of techniques such as microarray analysis, molecular sub-types were defined (2). Now, breast cancer is classified as hormone receptor positive based on the expression of Estrogen Receptor (ER), Progesterone Receptor (PR), and Human Epidermal growth factor Receptor-2 positive (ERBB2/HER2+) (3). Breast cancers that lack the expression of all 3 receptors are classified as triple negative breast cancers (TNBCs). Importantly, this molecular status defines the gene based therapeutic approaches presently employed in clinical practice for treating breast cancer (4, 5). About 70% of breast cancers diagnosed are positive for the expression of estrogen receptor (ER+) and sustained exposure to the hormone is known to cause cancer. Clinically, ER+ patients are treated with anti-hormone therapy leading to the development of a number of molecules that are selective estrogen receptor modulators (SERMs) like Tamoxifen (Tam), selective estrogen receptor degraders (SERDs), or inhibitors of the enzyme aromatase (AI), that converts androgens to estrogens (6, 7). Aromatase Inhibitors (AIs) are usually utilized as a second line of treatment in tamoxifen resistant tumors. Treatment with SERMs, in particular Tam, has decreased the mortality due to breast cancer by 25–30%. About 30% of women treated with Tam run the risk of recurrence in the next decade due to development of *de novo* resistance after prolonged exposure to the drug, especially in the metastatic setting. Intrinsic resistance to Tam is rare although single nucleotide polymorphisms in cytochrome p450 enzymes that affect the metabolism of Tam to its active form have been reported (8). This limits clinical benefit derived by the patients and two challenges emerge in the treatment of ER+ breast cancers, (1) availability of biomarkers that can predict endocrine resistance and (2) finding alternate agents to treat endocrine resistant tumors.

Few biomarkers exist to predict response/resistance to endocrine therapy. An exception to this is ER itself, and since ER negative (ER-) tumors rarely respond to Tam, presence of ER remains the most successful biomarker for response to endocrine therapy. However, cell culture data point to multiple other molecular mechanisms that may result in cells becoming refractory to estrogen inhibition (9). Interestingly, a significant portion of the cell culture findings have been correlated to Tam resistance in patients. Agents that interfere with these mechanisms offer the promise of novel therapeutic opportunities and are already used in combination with SERMs. In the review, we summarize data from candidate gene approaches and the more recent genomic studies that have revealed mechanisms leading to endocrine resistance.

### Mechanisms of Endocrine Therapy Resistance

Resistance developed during treatment may either be intrinsic, which is present in the individual before the start of any treatment, or the resistance is acquired during the course of treatment. Some pathways to acquired resistance are described below (9, 10).

#### Estrogen Receptor (ER)

Estradiol (E_2_) binds ER, a ligand activated transcription factor that interacts with palindromic estrogen response elements (EREs) located in the regulatory regions of its target genes to alter their transcription (11, 12). ER has two subtypes, ERα and ERβ, encoded by genes present in chromosome 6 and 14, respectively, which can homo- and hetero-dimerize to mediate their transcriptional action. Each ER subtype has a unique role in gene regulation, displays cell and tissue specific expression and alters different signaling pathways downstream (13). ERα activation promotes tumorigenesis since it induces proliferation and invasiveness of breast cancer cells. In contrast, ERβ appears to restrict cell proliferation, antagonize epithelial to mesenchymal transition and increase sensitivity to Tam in cell lines (14, 15). In patients, high levels of ERβ correlate with better survival and better response to Tam independent of ERα but lower levels of ERβ may contribute to endocrine therapy resistance (16, 17). In some reports, ERβ overexpression is observed in pre-invasive breast tumor of tamoxifen resistant individual and ERβ appears to have a negative effect on the transcription stimulated by ERα.

Further, both ER subtypes are alternately spliced and display differential gene regulation, cellular localization and pathogenicity in cancer (18). Atleast two truncated isoforms of ERα (ERα36 and ERα46) that arise due to alternate splicing, exon skipping and promoter usage act in a dominant negative manner as compared to the full length ER protein (19, 20). ERα36 localized to the mitochondrial membrane in uterine cells, performed non-genomic actions and was linked to Tam resistance whereas ERα46 was shown to increase sensitivity to Tam in Tam resistant MCF-7 cells. ERβ has multiple isoforms based on the exclusion of exon 8 such as ERβ1 (full-length), annotated as ERβ2 to ERβ5. Of these, nuclear ERβ2 and ERβ5 may antagonize ERα function but can enhance ERβ1 transactivation. Their relationship to endocrine resistance is still under investigation since they often are co-expressed with ERα (21). Due to such push and pull functions of ER subtypes, the ratio between ERα, ERβ, and their variants is being assessed as a predictive biomarker for endocrine therapy responsiveness (22). The subtype related data however remain controversial, mainly due to lack of good antibodies to differentiate between ERα and ERβ isoforms (23).

In addition to the nuclear activity, ERα can also localize to the plasma membrane and elucidate rapid non-genomic actions via activation of PI3Kinase/AKT and MAP kinase pathways, G-protein coupled receptor pathways or by changing calcium levels within the cells (24). Secondly, extranuclear ER activation is thought to occur via concentration of receptor tyrosine kinases, signaling proteins and ER in caveolae and lipid rafts (25). Such redistribution of ER is observed in Tam resistant cells, leading to its increased binding to EGFR and induction of downstream signaling. This likely describes yet another mechanism of endocrine resistance (26).

Given the substantial influence of ER on breast cancer biology it is logical to presume that mutations in ER and its partners may be the leading cause of intrinsic resistance to endocrine therapy. Loss of ER expression and the mutation in the estrogen receptor gene (*ESR1*) are the two important aspects of hormone based therapy resistance. However, in patients, mutations are rarely found in *ESR1* (<1% have mutations) or its associated proteins and deletion of ER accounts for only 10–20% of the cancer cases prior to therapy (27). In case of acquired resistance 17–28% of cases do not express ER (28). At times, the loss of expression of ER is due to aberrant methylation pattern at the promoter region or deletion of an exon (exon 5) in the ER mRNA (29, 30). The use of histone demethylase (HDAC) inhibitors like entinostat and Scriptiad and DNA methyl transferase (DNMT) inhibitors have been shown to restore the ER expression and hence sensitivity to tamoxifen treatment in breast cancer cell lines (31, 32). Some mutations in ER lead to hypersensitivity to circulating estrogen and make ER highly active such that tumors do not respond well to Tam therapy (33). Few tumors show *ESR1* amplification, no loss in ER expression but are resistant to Tam. These data suggest that numerous pathways beyond the expression status of ER may govern endocrine resistance.

#### ER Co-factors in Endocrine Resistance

ER is a modulator protein with N-terminal transactivation (AF1), DNA binding (DBD), a hinge, and a C-terminal ligand binding domains (LBD) containing a ligand induced transactivation domain (AF2). Bound ER is a complex of several co-regulatory proteins (co-activators/co-repressors), transcription factors and histone modifiers. Ligand bound ER adopts a conformation such that helix 3,5, and 12 along with AF2 form a hydrophobic pocket and a protein surface conducive to interaction with the leucine rich (LXXLL) motifs of co-activators- Tamoxifen is a competitive inhibitor of estrogen, binds the same regulatory sites as estrogen bound ER, but results in shifting the position of helix 12 away from the ligand binding pocket, such that Tam bound ER recruits a co-repressor complex instead of a co-activator complex to antagonize hormone response (34). Substitution of amino acids L536, Y537, D538 in this region positions Helix 12 in the agonistic conformation resulting in the constitutive transactivation of ER in absence of the ligand. Such and other missense mutations in this region of ESR1 are rare but described in acquired endocrine resistance and tumor metastases (35–37).

High expression levels of ER co-activators such as AIB1 (amplified in breast cancer 1) were shown to enhance the agonist activity of tamoxifen and contribute to tamoxifen resistance (38). Conversely, a progressive reduction in Nuclear receptor co-repressor 1 (NCoR) co-repressor activity during tamoxifen therapy enhanced the agonist effects of tamoxifen on ER contributing to resistance (39). ER also has non-classical genomic actions that involve indirect binding to DNA by tethering to other transcription factors such as Activator Protein (AP-1), Specificity protein 1 (Sp1), CCAAT/enhancer-binding protein beta (C/EBPβ), and cyclic AMP response element binding protein (CREB). Such complexes lead to the conversion of Tam-ER antagonist activity to an agonistic activity leading to proliferation of breast cancer cells (40). Enhanced expression of such factors also results in endocrine therapy resistance (41). For example, aberrant expression of other transcription factors such as SRY-box 9 (SOX9), a stem cell factor stimulated by the Runt-related transcription factor 2 (RUNX2)-ER complex promoted proliferation, metastatic phenotype and endocrine therapy resistance (42). Forkhead box protein A1 (FOXA1) is a pioneer factor that opens the compacted chromatin, facilitates the recruitment of ER to its genomic sites and co-expresses with ER during mammary gland development, and primary breast tumors development (12). FOXA1 is found to be over-expressed in endocrine resistant cell lines and it activates oncogenes and proteins like Interleukin-8 (IL-8) that are associated with endocrine resistance (43).

Reprogrammed chromatin landscapes that are independent of ER also drive endocrine resistance. Enhancer of Zeste 2 (EZH2), a methyltransferase that modifies chromatin, is found to confer endocrine therapy resistance by suppressing the expression of GREB1 which is a co-factor of ERα. The level of EZH2 was found to be high in tamoxifen resistant samples and a low level of GREB1 modifies the transcriptional machinery of ERα and a different transcriptome which provides refractory phenotypes in hormone positive cells (42). In another study, a significant difference between open chromatin landscape of sensitive and resistant MCF7 cell lines has been reported (44). No significant contribution of classical ERα signaling was observed in these cell lines. However, in the resistant cells, chromatin reprogramming led to enrichment and overexpression of the NOTCH pathway and other target genes in resistant cells, with the NOTCH3 pathway being responsible for the development of resistance. NOTCH3 induced Pre-B-cell leukemia transcription factor 1 (PBX1) expression and together they drove the expression of several endocrine resistance pathway genes (44).

#### ER and It's Crosstalk With Signaling Pathways

Often ligand independent ER activation occurs due to post translational modifications of the wild type ER protein. This is achieved by overexpression of growth factor signaling proteins that modify ER making the cell refractory to Tam action. The phosphorylation of ER is brought about through overexpression of various receptor tyrosine kinases such as HER2, Epithelial Growth Factor Receptor (EGFR), and Insulin like growth factor receptor (IGF1R) (45, 46). Phosphorylation renders ER active in a ligand independent manner making cells refractory to Tam action. Similarly, activation of MAPK or PI3K/AKT signaling pathway has been implicated in development of endocrine resistance (47). Further, in addition to phosphorylating ER, transfection of HER2 in hormone receptor positive cells led to the down regulation of ER expression conferring resistance to anti-hormone therapy (48, 49).

#### Role of miRNAs

MicroRNAs (miRNAs) are small non-coding RNA molecules of around 22 nucleotides in length that regulate the expression of various genes either by degrading their mRNA or suppressing their translation. miRNA 221 and 222 when expressed ectopically are found to convert the hormone sensitive MCF7 cell line to a resistant line by down-regulating a cell cycle inhibitor p27(Kip1) (50). This led to continued cell division despite the presence of ER blockers. These miRNAs were also found to be up-regulated in HER2 positive and ER- cells (51). Similarly, various miRNAs are found to be associated with hormone therapy resistance as their main targets are apoptotic genes or cell proliferation proteins and reviewed in more detail by Muluhngwi et al. (52).

#### Role of Extracellular Vesicles (EVs)

EVs/exosomes are 30–200 nm secreted particles that carry DNA, RNA, and protein cargo and are capable of transferring information and activities onto receptive cells (53). EVs isolated from the resistant MCF7 cell lines converted the sensitive cells to a hormone resistant type after 14 days of treatment and this process was irreversible (54). Transcriptional activity of ERα mediated via E2 was decreased; however there was no significant change in the protein level of ERα. EV treatment activated AP-1 and NF-κB transcription factors which were implicated earlier in hormone therapy resistance in breast cancer (55). Exosome mediated resistance is also achieved by the activation of PI3K/AKT pathway. Chen et al. further showed that EVs treated with RNase were not able to alter sensitivity of cells and that miR-100, miR-222, and miR-30a were responsible in the pathogenesis and therapy resistance of breast cancer (56, 57). Further, RNA contained within the EVs derived from the stromal cells transferred the resistance phenotype to the breast cancer cells. Therefore, both paracrine and juxtacrine signaling induced by EVs could lead to the development of a subpopulation of therapy resistant tumor initiating cells (58). Exploring the role of EVs may uncover additional mechanisms of resistance.

#### The Genomic Landscape of Endocrine Resistance

By candidate gene approach, copy number changes in cyclin D1 (*CCND1), ERBB2*, and *FGFR1* and mutations in the MAPK pathway have been associated with endocrine resistance (59, 60). However, since tumors evolve due to therapeutic pressure, comparing genomic aberrations of treatment naïve and post-therapy samples would aid the discovery of both intrinsic and *de novo* resistance biomarkers and identify additional targets for treatment. Genomic characterization of untreated breast cancer (The Cancer Genome Atlas, https://cancergenome.nih.gov/) has identified key drivers, stratified patients based on mutational profiles, and have emphasize the clonal heterogeneity of tumors (61). Deep and targeted sequencing using next generation methods can be used to catalog those rare mutations that may be present at low frequencies in clonal cells but get selected during the course of treatment. Early targeted sequencing of *ESR1* showed that mutations in this gene enriched in the LBDs (62). Whole exome profiling and RNA sequencing of 143 tumors from ER+/Her2- patients exposed to letrozole (AI therapy) identified intrinsic genomic alterations in *CCND1* and *FGFR1* genes and intrachromosomal *ESR1* fusion transcripts that could be responsible for endocrine therapy resistance (63). Next, a survey of mutations and copy number changes using a targeted approach to study 230 genes from ER+ tumors that were metastatic and progressed further while on endocrine treatment identified mutations in *ESR1* as well as Human Epidermal growth factor receptor 3 **(***ERBB3*) and Regulatory-associated protein of mTOR *(RPTOR)* (64). Mutations in *ESR1* clustered in the ligand binding domain such that the mutant receptors assumed an agonist conformation, had higher stability, increased interaction with co-activators or had higher transactivation function even in the absence of the ligand. Since patients resistant to SERMS are often treated with AI therapy, these data suggest that patients harboring such *ESR1* mutations may not benefit from this therapy as AI only decreases the availability of circulating estrogen levels in the patient. However, these mutants may be amenable to alternate SERMs. Using cell lines with stable expression of mutants, the authors demonstrated that SERMs could achieve 100% decrease in ER activity but only at extremely high doses. Therefore, development of more potent next generation SERMs may be required to diminish the risk of recurrence. Such mutants also appear to be more prevalent in metastatic disease and may possibly be causal in the process of tumor dissemination.

In addition to mutations in the ER protein, genomic structural rearrangements (REs) in the *ESR1* gene have been identified in recurrent metastatic ER+ breast cancer (65). The junctions were clustered between exons 6 and 7 of *ESR1* fused to unique partners on the 3′ end. Often such RE hotspots are also associated with a change in copy number (termed as copyshift). Most *ESR1* fusions or predicted REs may not be expressed or translated, but some had ligand independent activity and some fusions were hyperactive. Most endocrine therapies are designed to attack the LBD of ER, since *ESR1* REs commonly loose the LBD to gain fusion partners, they may drive endocrine resistance in patients and newer therapies blocking the N-terminal of ER are necessary.

Incidentally, mutations/fusions in *ESR1* account for only in 15–18% of patients that show endocrine resistance suggesting additional genes/pathways exist. A more recent genomic study on 1,501 HR+ tumors that characterize 809 therapy naïve and 692 tumors post therapy established a taxonomy of aberrations that associate with endocrine resistance (66). Functional hotspot mutations in *ESR1, ERBB2*, and loss of function mutations in Nuclear Factor 1 (*NF-1)* were found to be twice as common in post therapy samples as compared to therapy naïve tumors. Either rare clones with these mutations exist in therapy naïve individuals and they expand during the course of treatment or these may be *de novo* acquired changes. Interestingly, these events were mutually exclusive and non-overlapping. This study also identified mutations in the MAPK pathway in 16% of the cases and genes that regulate the expression of ER such as *FOXA1* and T-box transcription factor 3 (*TBX3*) were found altered in patients who did not have *ESR1* mutations. Interestingly, amplifications in genes like MYC and CTCF that also regulate ER expression were absent. Despite comparative and deep sequencing, these studies explain only 40% of the mechanisms in play and newer genomic mechanisms for resistance may exist.

## Conclusion

**Figure 1:**
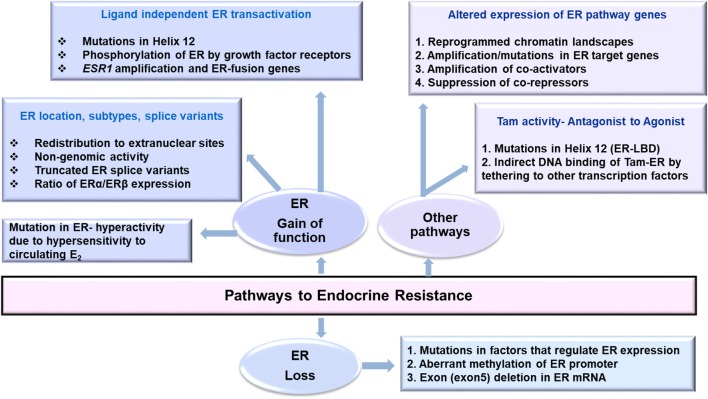
Pathways that may lead to endocrine resistance in Breast Cancer.

Changes in transcription of ER, its co-regulators, epigenomic, and post-translational modifications in ER, genetic polymorphisms affecting pharmacokinetics of anti-hormone drugs that affect ER expression, mutations in ER pathway that affect its activity along with therapy induced genomic aberrations may favor endocrine resistance (Figure 1). Owing to the complexity of ER biology in cancer, an integrative analysis of multiplatform data that can evaluate wellness trajectories during the course of treatment is necessary in identifying more mechanisms, and newer targets to combat endocrine resistance. A major challenge is the availability of matched pre-and post-therapy samples in sufficient amounts to perform all analyses simultaneously. Longitudinal sampling to monitor disease progression would be ideal to determine tumor relapse and recurrence but such samples remain unavailable. Here better and highly sensitive methods to analyze liquid biopsy samples such are circulating cell free DNA (ctDNA/cfDNA) and contents of EVs are emerging (67). How closely they resemble and represent tumor evolution, clonality of tumor cells, characteristics of disseminated disease, and how well they can predict response to endocrine therapy remains to be seen.

## Author Contributions

Both MH and KD contributed toward the writing and editing of this review.

### Conflict of Interest Statement

The authors declare that the research was conducted in the absence of any commercial or financial relationships that could be construed as a potential conflict of interest.
